# Need for information, honesty and respect: patient perspectives on health care professionals communication about cancer and fertility

**DOI:** 10.1186/s12978-017-0441-z

**Published:** 2018-01-05

**Authors:** Jane M. Ussher, Chloe Parton, Janette Perz

**Affiliations:** Translational Health Research Institute, School of Medicine, Western Sydney University, Locked Bag 1797, Penrith South, 2751 Australia

**Keywords:** Cancer and fertility, Infertility, Parenthood, Gender differences, Health care professional communication, Treatment satisfaction, Fertility preservation, Health information

## Abstract

**Background:**

Individuals affected by cancer report a need for information about fertility from health care professionals (HCPs), in order to inform decision making and alleviate anxiety. However, there is evidence that many health professionals do not engage in such discussions.

**Method:**

A mixed method design was used to examine the construction and subjective experience of communication with health professionals about fertility in the context of cancer, from the perspective of patients. A survey was completed by 693 women and 185 men, across a range of cancer tumour types and age groups, and in-depth one-to-one interviews conducted with a purposively selected subsample of survey respondents, 61 women and 17 men. The chi square test for independence was used to test for group differences between women and men on closed survey items. Thematic analysis was used to examine the open ended survey responses and interviews.

**Results:**

Significantly more women (57%, *n* = 373) than men (46%, *n* = 80) (*X*^2^_(2517)_ = 6.54, *p* = .011) reported that they had discussed fertility with a HCP since diagnosis of cancer. Satisfaction with the discussion was reported by 65% (*n* = 242) of women and 69% (*n* = 54) (*ns)* of men. This discussion was reported to have been initiated by the patient or their partner in 44% (*n* = 165) of women and 47% (*n* = 37) (*ns*) of men. In the interviews and open ended surveys three themes were identified: Feeling heard and informed about fertility after cancer: Positive experiences of HCP communication; “I was never given full disclosure”: HCP silence or reticence about discussing fertility after cancer, including the sub-theme “Their primary concern is getting me cancer free”: Constructions of absence of fertility communication by HCPs; and Confusion and lack of compassion: Unsatisfactory information provision about fertility and cancer.

**Conclusion:**

Discussion with a HCP about fertility concerns, and satisfaction with the discussion, was associated with reports of lower patient distress, greater knowledge and understanding of the consequences of cancer on fertility, involvement in the decision making process about fertility preservation, and satisfaction with health care.

## Plain English summary

Individuals affected by cancer report a need for information about fertility from health care professionals (HCPs), in order to inform decision making and alleviate anxiety. However, there is evidence that many health professionals do not engage in such discussions. This study examined the experience of communication with HCPs about fertility from the perspective of 693 women and 185 men patients, across a range of cancer tumour types and age groups. Women were more likely than men to report that they had discussed fertility with a HCP since diagnosis of cancer (57%, *n* = 373 women; 46%, *n* = 80 men). Satisfaction with the discussion was reported by 65% (*n* = 242) of women and 69% (*n* = 54) of men, with discussion initiated by the patient or their partner in 44% (*n* = 165) of women and 47% (*n* = 37) of men. Participants who engaged in discussion with a HCP about fertility concerns, and who were satisfied with the discussion, had greater knowledge and understanding of the consequences of cancer on their fertility, felt that they were involved in the decision making process about the timing of treatment, and were able to discuss options for fertility preservation, where appropriate. In combination, this served to increase health literacy associated with cancer related infertility, providing the sense of self-efficacy that is essential in coping, in order to reduce the distress and threat to identity that cancer related infertility can produce. This confirms that discussion of fertility concerns by oncology clinicians is “a crucial aspect of high quality healthcare”.

## Background

There is growing evidence that compromised fertility can be one of the most difficult long term effects of cancer treatment [1], associated with depression, anxiety, grief, low self-esteem, and changes to body image and gender identity [2–5]. Individuals affected by cancer report a need for information about fertility from health care professionals (HCPs), in order to inform decision making and alleviate anxiety [6–9]. Levels of fertility related distress are lower in individuals who have received pre-treatment information from HCPs about the impact of cancer on fertility [10], counselling about options for fertility preservation [11], and who are satisfied with the information provided [5, 7].

Advances in fertility preservation options have allowed fertility to be addressed at earlier stages in cancer care [12, 13]. Indeed, discussion of fertility concerns by clinicians has been described as “a crucial aspect of high quality healthcare” which helps with patient adjustment [14] , p. 126. Clinical guidelines [15, 16] and researchers [17–19] recommend that fertility information be provided at the point of diagnosis. As infertility can be a late effect of cancer [20], information is also needed after treatment has ended. However, there is evidence that many health professionals do not engage in discussions about fertility after cancer [3, 6, 7, 17, 21]. This may be because of lack of knowledge, time constraints, personal discomfort [22, 23], or because they position it as too difficult, or not relevant, with particular patients [17, 21, 24]. As a result, a significant number of cancer patients report that they received no information about fertility, or cannot remember what they were told [25]. The proportion of patients who received no fertility information ranges from 20% [26] to 62% [27] of individuals surveyed across studies, the average being around 50% [28–30].

Fertility information is more likely to be provided to younger people [31], reinforced by fertility guidelines which focus on adolescent and young adults (AYAs), which can result in the neglect of fertility concerns for older adults [30]. Men are more likely than women to report discussion of fertility with a health professional and to report satisfaction with the discussion [25, 32–35]. This is a matter of concern as women rate the need for fertility information and services more highly than men [36], and are more likely to be distressed about the possibility of compromised fertility [1, 32, 37, 38]. Women are more likely than men to report negative experiences of health care professional communication about fertility [32, 39]. Some women report that they feel robbed of a choice by the health system, due to lack of information and advice about fertility preservation [40]. Fertility discussions can also be difficult for men. Sperm banking is associated with reports of embarrassment [41, 42], or with being rushed after diagnosis, with little regard for the emotional impact of the process [43, 44].

One of the limitations of previous research on fertility and cancer is that it has been conducted from a medical perspective, with little attention being paid to the “reproductive motivations” of cancer survivors [45], p. 6, the psychosocial concomitants of fertility concerns [46], or the gendered nature of compromised fertility and interactions with health care professionals [35]. Previous research has also been criticised for being small scale, with participants primarily recruited from a single clinical site, and focusing on a one tumour type - primarily cancers that affect the sexual organs [9, 35]. There is evidence that a wide range of cancers and cancer treatments may impact upon fertility [7, 47], and that fertility related distress does not differ across tumour type [4]. This suggests a need for a more comprehensive study across a broad range of cancer types and clinical sites, to examine the experience of patient-health care provider communication about cancer related fertility concerns.

The aim of this study was to examine the construction and subjective experience of communication with health professionals about fertility in the context of cancer, from the perspective of women and men cancer survivors, across a range of cancer tumour types and age groups. Our research questions were: Are there differences between women and men in the extent and satisfaction with health professional communication about fertility? How do men and women construct the discussion of fertility with health professionals, and what are the reported consequences for subjective wellbeing?

## Method

### Procedure

This study was part of a mixed-method project which examined the construction and experiences of fertility after a cancer diagnosis. Participants responded to advertisements circulated nationally through cancer support groups, social media, media stories in local press, advertisements in cancer and carer-specific newsletters, hospital clinics, and local Cancer Council Websites and telephone helplines. Participants completed an online or postal survey examining their experiences of fertility and infertility post-cancer. At the end of the survey, participants indicated whether they would like to be considered to take part in an interview, to discuss changes to fertility in more depth.

The survey included a series of closed and open ended questions about fertility and cancer. In this paper, we focus on participant responses to a series of items associated with communication with health professionals: Since receiving the cancer diagnosis have issues about fertility been discussed with a health care professional? How satisfied were you with the discussion? Have you received or used information or resources on cancer and fertility?

Interviews were conducted one-to-one by telephone, taking approximately 1 hour, and were digitally recorded and transcribed verbatim. The topics covered in the interview included: feelings about fertility and parenthood, the influence of fertility issues on romantic relationships, changes to personal identity and body image since being diagnosed with cancer, and experiences of interacting with health professionals. The interviews were conversational in style, with the wording and formatting of questions used flexibly to suit the particular context of the participant [48]. Participants were given a modest reimbursement for expenses, in the form of a gift voucher for $25 (AUD). All the interviews were transcribed verbatim by professional transcribers, and integrity checked for accuracy by a member of the research team.

### Analysis

The chi square test for independence was used to test for group differences between women and men on closed ended survey responses. Valid percentages are presented in the reporting of each survey item. Thematic analysis [49] was conducted using an inductive approach, with the development of themes being data driven, rather than based on pre-existing research on fertility and cancer. This process involved researchers reading through the responses to each interview in order to identify first order codes such as ‘negative experiences’, ‘positive experiences’, ‘fertility information offered or not offered’, and ‘information, support and resources’. The entire dataset was then coded using NVivo, a computer package that facilitates organisation of coded qualitative data. All of the coded data was then read through by a member of the team. Codes were then grouped into higher order themes; a careful and recursive decision making process, which involved checking for emerging patterns, for variability and consistency, and making judgements about which codes were similar and dissimilar. The thematically coded data was then collated and reorganised through reading and rereading, allowing for a further refinement and review of the themes, where a number of themes were collapsed into each other and a thematic map developed.

## Results

### Participants and themes

Eight hundred and seventy-eight people living with cancer (693 women, 185 men) completed the survey. The average age of survey participants was 42.53 years (SD = 14.21), and average time from diagnosis 6.22 years (SD = 7.01). The sample was drawn across cancer types including breast (56.7%), gynaecological (12.9%), hematologic (12.7%), gastrointestinal (4.8%), neurologic (3.2%), head and neck (2.9%), skin (2.3%), musculoskeletal (2.3%), genitourinary (0.9%) and respiratory (0.7%). Disease diagnosis status ranged between early and advanced stages, with 67% reporting that their cancer was diagnosed at an early stage. The sample was almost exclusively heterosexual (98%), with 71% reporting that they were currently in a relationship. Fifty-seven percent of survey respondents reported that they had a child.

256 people living with cancer (199 women, 57 men) indicated they would be willing to participate in a follow up interview. Purposive sampling [50] was used to select interview participants who had expressed concerns about fertility after cancer, with the aim of gaining insight into the experience of people across gender, age groups, cancer type, relationship contexts, and parenthood status (parous/nulliparous). Seventy-eight participants aged between 18 and 58 (*M* = 45.10), 61 women and 17 men, accepted the invitation to take part in the interviews.

The final themes developed from the analysis of the interviews were**:** ‘Feeling heard and informed about fertility after cancer: Positive experiences of HCP communication’; ‘“I was never given full disclosure”: HCP silence or reticence about discussing fertility after cancer’, including the sub-theme ‘“Their primary concern is getting me cancer free”: Constructions of absence of fertility communication by HCPs’; and ‘Confusion and lack of compassion: Unsatisfactory information provision about fertility and cancer’. In the presentation of results, gender, age and cancer type is indicated for longer quotes.

### Feeling heard and informed about fertility after cancer: Positive experiences of HCP communication

In survey responses, significantly more women (57%, *n* = 373) than men (46%, *n* = 80) (*X*^2^_(2,517)_ = 6.54, *p* = .011) survey respondents reported that they had discussed fertility with a HCP since diagnosis of cancer. Of those who reported discussing fertility with a HCP, 65% (*n* = 242) of women and 69% (*n* = 54) (*ns)* of men were satisfied with the discussion. Explanation for satisfaction was constructed in terms of HCP’s being “proactive” in informing participants about the possible consequences of cancer treatment on fertility, as well as being “informative,” “clear, accurate,” and “explaining everything well.” Information and advice from HCP’s allowed participants to make “informed decisions” regarding their cancer treatment and consequences for fertility. For example, one woman (34, Gynaecological) said that in her experience, HCP discussions “help you understand the medical side of the cancer and how it affects you”. Many participants valued “honesty”, reporting preferences for comprehensive information in instances where treatment would likely result in detrimental fertility outcomes. For example, as one woman said “they always explained everything well, including the possibilities of being infertile in the future – they didn’t try to sugar coat anything” (19, Ewings Sarcoma). Another woman said that it was “satisfying to get a clear view of what was possible and not possible” (49, breast).

Accounts of positive interactions with HCPs extended beyond the content of fertility information, to include the manner in which it was delivered. Participants described positive encounters with HCP’s as those where they felt that HCP’s employed “acceptance,” “warmth”, “understanding,” “sensitivity,” “care,” “respect,” “empathy,” “compassion” and had “taken the time to listen.” In these accounts, HCP’s were described as acknowledging how “serious” fertility was and did not “minimise things,” as evidenced by the following account.

I liked that the fertility doctor – she didn’t mess about. She told me the facts. I’d rather hear the facts than someone say, “Well there is a chance,” but then try and put a positive on it. I just want to know the facts and what my chances are, rather than have someone try and protect my feelings (female, 26, breast).

In a similar vein, a man (48, bowel) reported that “the doctors were great. They treated me with respect and I suppose honesty as well”. These positive experiences with HCP’s were reported to have left participants feeling “understood” or “heard” because they had been given time to voice their feelings and concerns. For example, participants told us that they had “come away from the appointment relieved and less stressed” (female, 23, leukaemia), and that they valued being “given the opportunity to verbalise how I was feeling” (female, 50 years, breast).

Many participants gave accounts of valuing written information as part of their consultation with HCPs about fertility concerns. In response to the survey, 46% (*n* = 298) of women and 51% (*n* = 49) (*ns)* of men indicated that they had received or accessed information resources regarding fertility and cancer. Examples included being given a “pamphlet,” “booklet” or “book”, with two participants commenting: “they would recommend resources and things to me” (female, 35, breast) and “I’m sure I was handed a lot of written information” (male, 40, Hodgkin’s Lymphoma). It was reported that this “made my life easier”, as a man told us: “I mean, I didn’t have to go out and search for it, she knew exactly sort of what things I was looking for and she’d be able to provide those to me” (24 years, Ewings Sarcoma). Another participant commented on the value of written information in supplementing medical consultations saying, “what you take in during a consultation, a face to face consultation, you might miss a lot of things” (female, 43, breast). Participants also spoke positively about instances in which written material was provided by HCP’s who then “went through everything” with participants.

Willingness of HCPs to discuss fertility preservation, and make a referral if appropriate, was also reported to be a positive experience, allowing individuals to “know what my options are and then make a decision” (female, 32, breast). Participants valued being given the “choice”, “opportunity” and “time” to fully investigate fertility preservation options, enabled through prompt referral by HCP’s, and where possible, timing of cancer treatments to accommodate fertility procedures: “they were more than happy to write referrals to the relevant fertility specialist” (female, 44, breast) and “I was pleased that the surgeon said that this could be a possibility before I agreed to have surgery” (male, 41, brain). Some participants described HCP’s as supportive in the decision making process about fertility preservation services, without attempting to influence the outcome. For example, “my GP was fantastic, as were all the staff at our health centre. She never tried to influence us, but said she would support us whichever route we decided on” (female, 45, Gynaecological).

### “I was never given full disclosure”: HCP silence or reticence about discussing fertility after cancer

Of those participants who had not discussed fertility with HCP’s, 43% of women and 64% of men, the majority constructed the experience in terms of absence of information: “I was never given full disclosure of side effects of treatment” (female, 32, gynaecologic), “there was no expert opinion on what to do” (male, 37, testicular), and “nothing was offered by anybody” (female, 41, breast). The consequence of absence of information was positioned as lack of knowledge on the part of patients of the fertility consequences of cancer treatment. For example, a number of men said that they did not know that treatment “could make me sterile” (20, osteosarcoma), which was a regret as “I honestly didn't know and I wish that I had” (male, 41, brain). Similarly, a number of women told us “I wasn't aware that the chemo would affect fertility” (27, non-Hodgkin’s Lymphoma) and “I didn't realise my womb was going to go. I think he should have told me before it happened” (36, gynaecological). Participants described the lack of fertility discussion by HCP’s as contributing to psychological distress associated with compromised fertility, which was described as “heartbreaking”, “sad” and a “loss” that led to “grief”. One interviewee (Charlotte, 41, breast) talked about how the lack of fertility information contributed to a more challenging cancer experience, saying, “when you do have all the information, you can then go into something with your eyes open, whereas to find out things after the fact that it - it makes it so much harder to handle.” Another women (34, breast) told us that she “felt abandoned” by being left in the dark about the impact of cancer on her fertility.

Absence of information was associated with lack of access to decision making or interventions to preserve fertility, with participants saying, “we didn’t really get a choice” (female, 31, breast), or that fertility was “never in question at all and no alternative offered” (male, 48, bowel cancer). Subsequently, many participants reported finding out about their impaired fertility after the conclusion of treatment, when they had diminished options for intervention. As one man commented: “It was sort of like you need this (treatment), and that’s happening, blah, blah, blah and then it wasn’t until afterwards we thought ‘well hang on what about fertility?’” (male, 37, testicular). Another woman (32, throat) said, “I have been told that I will not be able to naturally produce children, if I was made aware of this when I was first diagnosed I would have stored some eggs.”

In the absence of fertility information being offered by HCPs, participants positioned themselves as responsible to source information in order to “understand what the hell had gone on with me” (male, 37, testicular), or to “not panic, and work out ‘where do I go to from here?’” (female, 43, gynaecologic). Such information was also described as having helped participants to be more prepared for their interactions with HCP’s, evidenced in the following accounts: “I was relatively aware … I’d been doing my own research” (male, 26, Hodgkin’s Lymphoma); “I do believe very strongly in getting a lot of information because then you can ask the right questions as well” (female, 35, breast). Sources of written information included online websites and blogs, and booklets produced by cancer organisations, supplemented by verbal discussion facilitated by community organisations, such as support groups and workshops. Such information was not positioned as a substitute for discussion of fertility with HCPs, however, with some participants describing written information as “worrying”, “overwhelming”, “not in-depth enough”, or “too general”. The majority of participants wanted written information combined with discussion of their specific concerns with a HCP, illustrated by Louisa’s (19, gynaecologic) account below:

I would have preferred to get the information from my doctor. To sit down and ask the questions and him tell me all of my options and then give me the leaflets and be like, ‘we’ve discussed what options you’ve got for the future, I’ve gathered a few different resources if you wanted to do a little bit of home research and look into a few of them’.

In the absence of HCP communication about fertility, many participants took up a position of responsibility for initiating the discussion. In survey responses, 44% (*n* = 165) of women and 47% (*n* = 37) (*ns*) of men who had engaged in discussion with HCPs about fertility reported that they or their partner were the one to raise the issue. This was explained in qualitative accounts, with one woman telling us: “I feel as though they [HCPs] are waiting for me to explain how I feel about infertility before they comment” (36, breast). In many instances, participants described having to “push” HCP’s in order to access information, or to be referred to a fertility specialist: “it’s just that I had to mention it two or three times rather than once” (female, 32, breast), and “it just felt like I had to push for it sometimes” (female, 34, breast). Access to fertility preservation was also described as having occurred due the initiative of the patient, rather than the HCP: “it was only because I asked that I ended up getting help and freezing the embryos” (female, 42, breast). This was reported to have made access to fertility information and interventions more “challenging”, with some participants describing difficulties in finding specialist care or information. As one woman commented: “I struggled to find anyone knowledgeable about fertility preservation in breast cancer cases” (41, breast).

*“Their primary concern is getting me cancer free”: Constructions of absence of fertility communication by HCPs*

Participants made sense of HCP reticence to discuss fertility in a number of ways. Some participants attributed the absence of information to HCP focus on treatment and survival, with a number of women participants reporting feeling rushed into treatment, rather than having the opportunity to address fertility concerns: “[the] GP was more concerned with initiating treatment” (female, 37, breast); “their primary concern is getting me cancer free” (female, 31, breast). Participants’ also spoke of being treated by HCP’s who appeared to lack knowledge about the ways in which cancer treatment might compromise fertility, or knowledge of the fertility options available. As one woman (37, breast) said, “we felt none of the medical professionals had a strong understanding of the impact of treatment on fertility and the options that were available”. Another man (27, thyroid) said,

I just think the data is not there anywhere for them to raise. And doctors typically I found that are cautious, but if they don’t have a bit of information in front of them, then they don’t discuss it.

A number of participants described feeling that their HCP experienced “discomfort” at having fertility concerns raised. For example, “that wasn’t something he wanted or probably knew a lot about. I don’t think he felt comfortable discussing that at all” (female, 41, breast). Another woman was treated for cancer as a child and described fertility as a “taboo,” amongst paediatric HCP’s. She told us that her mother raised fertility concerns at diagnosis “and the doctors were just speechless at the fact that she openly spoke about the issue” (19, Leukaemia).

A number of participants, primarily women, gave accounts of feeling that HCP’s made inaccurate assumptions about their need for information about fertility, as one woman said, “sometimes professionals think they know what is best for you without asking you” (38, breast). It was reported that HCP’s made assumptions based on whether or not a woman currently had a partner, with participants saying, “often people dismiss your feelings because you aren't in a relationship, so to them you mustn’t have been thinking about children anyway” (44, endometrial). Participants also reported feeling that HCP’s made assumptions based on their “older” age at the time of diagnosis, for example: “I feel that due to my age and the fact that people seem to feel that I had already made a distinct choice not to have a child earlier (it wasn’t an issue). That's not really the case at all” (49, breast). Conversely, some young adult participants reported feeling that fertility options were “overlooked” due to their younger age at diagnosis, or that discussion that did take place was directed at their parent, as one woman (24, haematologic) told us:

And I felt like the doctor was talking more to my mum than to me. I mean, I was 21, I was an adult, I’d lived out of home for four or five years and I still felt like they were treating me a little bit as a child.

Other women spoke about feeling fertility concerns were overlooked by HCPs because they already had a child, saying: “Oh she’s already got one [baby] so we don’t need to talk about this” (female, 41, breast), and “perhaps if I hadn’t had any children, then they might have come forward with more support” (female, 35, breast). Across the board, participants were in agreement about the importance of HCP’s not making assumptions and providing information and fertility preservation options “whatever age they may be,” regardless of current relationship and children.

### Confusion and lack of compassion: Unsatisfactory information provision about fertility and cancer

A substantial proportion of participants who engaged in discussion with HCPs did not report satisfaction with the discussion, 35% (*n* = 129) of women and 31% (*n* = 24) of men. A number of explanations were provided for unsatisfactory discussions. Many participants reported receiving inaccurate or conflicting information from HCPs regarding their fertility after cancer, leading to “confusion” and feelings of “frustration”. For example, the following comments were made: “I found the process confusing, rushed, bewildering and confronting” (male, 18, Lymphoma), and “it is confusing and concerning to have so many different views” (female, 30, breast). “Conflicting information” was associated with reports of “difficulty” in understanding the impact of cancer treatment on fertility, and “uncertainty” regarding “how long to wait” and the consequences of treatment for conception and pregnancy; “no-one ever told me clearly how safe or unsafe it would be for me to have another pregnancy (i.e. whether the hormonal changes in pregnancy could put me at risk of more cancer)” (female, 46, breast).

In addition to conflicting information, participants gave accounts of dissatisfaction due to “limited information”, lack of “real discussion,” or conversations that did not acknowledge the “importance” or “priority” of fertility concerns. For example, one woman (43, breast) said she received information in an “itsy, bitsy kind of way,” and as a “throwaway comment said while we were standing up waiting for something. It wasn’t even at his desk or anything.” Another woman said fertility options were “lightly glossed over” and a man commented: “I was only given a brief run down about having radiation and how you never regain fertility” (21, leukaemia). Lack of “in depth” communication was reported to have contributed to psychological distress in participant accounts. For example, a brief comment about fertility control left a woman melanoma survivor “distraught” because it led to her realisation that she had a poor prognosis: “It was probably the first time I realised I wasn't expected to live. The doctor said ‘no children for five years’ and I asked ‘why?’ and she said ‘young children needed a mother’” (female, 40, Melanoma). Participants also described difficulty processing information when fertility was discussed “in passing”, contributing to challenges for decision making. For example, a number of participants gave accounts of infertility only being mentioned immediately prior to treatment commencement. As one women said,

I was asked the question on the day before I started chemo, that was I concerned about my fertility and did I wish to postpone to participate or to look at fertility options. That was pretty much all I was really given or spoken to about (28, respiratory).

In addition, some male participants also did not understand fertility preservations options when they were presented, contributing to “confronting” experiences, as exemplified by one participant who said,

they just sort of were like, “Okay, yep, well this is the issue, you go do your sperm banking and then we’re done, we’ll, we’ll start treatment the week after that.” Then it’s sort of like, “Whoa, whoa, whoa, wait, why am I sperm banking?” (male 24, Ewings Sarcoma)

A further source of dissatisfaction for a number of participants was perception of negative responses from HCP’s when the patient or their partner raised fertility concerns. HCP’s were described as being “dismissive”, “unsympathetic”, or lacking in “compassion” or “empathy” in response to participants requests for information or referral, reporting: “they didn’t seem to care” (male, 47, testicular) and “I felt a lack of personal touch when I was told about our situation” (female, 39. Hodgkin Lymphoma). These dismissive interactions with HCP’s were described as contributing to feelings of psychological distress. As one woman said, her HCP was “really dismissive” and “blunt.” She described feeling there was “no hope presented,” and that “it really did have a big impact on my mental health and emotional health” (female, 35, breast). Other participants were distressed with the “clinical” nature of fertility information provided by HCPs: “I appreciated the honesty, but the trauma of the whole cancer experience was still very real and the clinical, cold answers were a bit upsetting” (male, 42, testicular). Many participants told us that they felt that the emotional gravity of fertility was not recognised by HCP’s involved in their cancer care. As one woman said,

Like, it was all quite, you know, they were all just sort of trying to distract us and being quite cheerful. And – but it just felt really invalidating and really painful to be the only person that was aware of what this chemotherapy meant to me. I’d like for everybody who’s involved in these treatments that can affect our fertility, to be aware of the emotional impact of these treatments on us. They should be sensitive (female, 36, breast).

Women described interactions with HCP’s where they felt they should be grateful for survival and nothing more. For example, “the psychologist I saw was unsympathetic - you are alive, why do you care about fertility/attractiveness etc” (female, 39, breast)’; “I was told that my focus was to survive and anything more than that would be considered a bonus. I was 19” (female, 23, leukaemia).

Many participants acknowledged that the focus of HCP’s on survival was understandable, given the emphasis within the health system on cancer cure. However, it was also argued that other aspects of the person, including fertility, should also be considered. As one participant commented, “cancer doctors should be more prepared that they are treating a human being with hopes and ambitions, not just a disease” (female, 45, breast cancer).

## Discussion

The findings of study support previous research which identified information about the impact of cancer on fertility as a priority for patients who are AYA or of reproductive age, serving to alleviate distress and improve quality of life [6–10, 25, 30]. Participants in the present study who engaged in discussion with a HCP about fertility concerns, and who were satisfied with the discussion, had greater knowledge and understanding of the consequences of cancer on their fertility, felt that they were involved in the decision making process about the timing of treatment, and were able to discuss options for fertility preservation, where appropriate. In combination, this was reported to increase their knowledge and confidence in discussing cancer related infertility, providing a sense of self-efficacy essential in coping [51–53] and alleviation of the distress and threat to identity that cancer related infertility can produce [2–5].

Increased knowledge and confidence in discussing health concerns through information provision is known to be associated with positive health outcomes [54]. More specifically, counselling about options for fertility preservation pre cancer treatment is associated with lower fertility related distress and regret [10, 11], and greater psychological wellbeing [55, 56]. Fertility counselling is also associated with ‘repaired’ identity and the opportunity to engage in fertility preservation [57]. This is confirmed in the qualitative accounts in the present study, with reports of acceptance of compromised fertility more prevalent in those who had the option to discuss fertility preservation with HCPs, even if it was not taken up, and fertility preservation associated with a sense of hope for the future. This illustrates the importance of individuals with cancer being given information about the impact of cancer on fertility [9, 12, 25, 39], and having the opportunity to engage in discussion about fertility preservation pre-treatment, if this is appropriate [9, 40, 58]. Previous research has reported that use of a fertility related decision aid (DA) can reduce decisional regret and improve satisfaction with the information received on the impact of cancer treatment on fertility [59], which suggests that the presentation of fertility information in a systemic manner is beneficial for patients. However, a recent review of clinician provision of oncofertility support found that the majority of oncologists rarely offer this material to patients, and when material is provided, it is often not age appropriate [25].

Participants who received information and advice about fertility from HCPs in a manner that was honest and respectful were more likely to report satisfaction with health care, and with the outcome of fertility preservation discussions, as a result of feeling that their concerns were acknowledged and questions addressed. It is widely recognised that positive and empathic doctor-patient communication is a vital part of cancer care, which can result in greater satisfaction with treatment [60, 61]. Treatment satisfaction, in turn, is associated with higher quality of life in cancer patients [62]. The findings of the present study therefore suggest that HCP communication about fertility concerns is not only important in relation to alleviation of fertility related anxiety, as suggested previously [10, 27, 57], but will also influence the clinician-patient relationship [56], with implications for the wellbeing of the patient.

HCPs are known to avoid discussing fertility with patients who have a poor prognosis, or for whom they believe parenthood is not a concern, due to factors such as relationship status, sexual orientation, or age [21, 23, 24, 63], as was reported by many participants in the present study. This resulted in the potential marginalisation of the fertility concerns of a range of patients, the majority being women. These patients may not be considering parenthood in the near future, and may not have a strong chance of a viable future pregnancy, but still wish to be informed about the possible options, and want HCP to avoid making assumptions about their desire to discuss fertility [40, 58]. Previous research has suggested that HCPs are influenced by personal assumptions in their interactions with patients [24], and that discussions about fertility with cancer patients are a difficult process [64–67]. This provides some explanation for why the recommendations of clinical guidelines which prescribe discussion of fertility with AYA cancer patients and those of reproductive age are not always followed [21]. However, the level of patient distress and dissatisfaction with care associated with absent or unsatisfactory information provision suggests that HCPs need to overcome personal difficulties in providing such information, and avoid making inaccurate assumptions about a patient’s desire for future parenthood.

It has been reported that men are more likely than women to report discussion of fertility with a health professional, and are also more likely to report satisfaction with the discussion [25, 32–35]. This was not supported by the findings of the present study, where women were significantly more likely to report having discussed fertility with a HCP. This finding may reflect increased awareness of the importance of fertility on the part of HCPs working in oncology, particularly breast cancer – the largest group of participants in the present study. However, a high proportion of HCPs are clearly not adhering to clinical guidelines that advocate fertility information provision [15, 16], as approximately 50% of participants had not engaged in discussion with HCPs, a proportion comparable to other recent studies [25, 28–30]. This indicates that a substantive number of individuals were not informed about the possibility of infertility following cancer treatment, across gender, resulting in unmet information needs and fertility related distress [9, 27, 28, 35]. This was manifested by clinicians not providing any information about the consequences of cancer treatment for fertility prior to treatment, HCP reticence in discussing fertility concerns when they were raised by the patient, limited or conflicting information provision by HCPs, and communication that was lacking in respect or empathy. This indicates that that even when HCPs do follow clinical guidelines [15, 16] and mention fertility, it may not be in a manner that meets that patient’s needs. Many individuals sourced their own information about fertility, and used this to initiate discussions with HCPs, or to supplement limited communication with HCPs, as has been reported previously [8, 68]. This demonstrates agency and self-efficacy on the part of patients, and can be a positive aspect of coping with cancer [69, 70]. However, the majority of participants in this study also wanted a detailed discussion with HCPs, and were disappointed and distressed by its absence or inadequacy.

These findings have a number of implications for clinical practice and policy. Reports of absence of discussion of fertility concerns by a substantial proportion of clinicians suggest a deficit in institutional policy and practice guidelines to assist HCPs with such discussions [21]. It has previously been reported that no single professional group possesses all of the skills or information necessary to effectively address the complexity of fertility concerns after cancer [71], and current clinical guidelines do not stipulate whose role it is to provide oncofertility care [21]. Agreement on a standardised referral pathway to assist in the provision of such care [24], Many HCPs working in oncology have a low level of knowledge about fertility preservation options or appropriate facilities [71]. There is also evidence that many HCPs hold negative beliefs about the importance of fertility for their patients [21], as reported in the present study. Additional training is needed to address such beliefs and to equip HCPs with skills to address oncofertility concerns [72–74], and many clinicians report interest in such training [21, 24]. In particular, it is vital that physicians providing cancer treatment should be aware of the effects of treatment on fertility and of ways to minimise these effects [75].

As the psychosocial experience of infertility has been reported to be different across genders [76, 77], with higher levels of fertility related distress reported in women cancer survivors [1, 32, 38], it is important that fertility information is gender specific, and sensitive to the gendered concerns of patients and their partners [76]. This includes awareness of the potential impact of compromised fertility on the gender identity of women [57, 78] and men [79, 80], and the gendered experience of fertility preservation, including difficulties in the collection of sperm [17], or the experimental nature of fertility preservation, when IVF is not possible, for women [81]. It has been argued that there is a need to bridge the gap between the two separate disciplines of oncology and fertility preservation, through the creation of multidisciplinary teams that include oncologists, nurses in the specialities of oncology and infertility, social workers, reproductive endocrinology and infertility specialists, andrologists, and embryologists, working together to improve the outcomes for cancer survivors [82]. Continued education about the rapidly changing field of fertility preservation techniques [81], as well as peer support and consultation [24], would be suitable in such a context. However, clarification of whose role it is to address oncofertility within a multidisciplinary team, is needed, as not all members of a team can be expected to raise the issue [21]. There is evidence that lack of availability of fertility counselling and fertility preservation services can act as a barrier to referral and utilisation of services [21]. This suggests that further efforts need to be made to raise awareness of the importance of oncofertility for service providers and policy makers, who make the decisions about resource provision within health systems.

This study had a number of strengths and limitations. The strengths were the use of a survey of a relatively large sample of men and women, across cancer types and age groups, and qualitative interviews to examine subjective accounts of infertility in depth. The limitations include the fact that the sample was not nationally representative, and that participants were recruited as part of research study examining experiences of fertility after cancer, which may have resulted in a greater focus on infertility within the accounts, and the greater proportion of women in the sample. The predominance of women and heterosexual cisgender individuals in the sample is also a limitation. Further research using a more equal sized sample of women and men, including a greater proportion of LGBTI individuals, is needed to examine the experience and impact of communication about fertility with HCPs following diagnosis of cancer.

## Conclusion

In conclusion, compromised fertility following diagnosis and treatment for cancer can have a negative impact on psychological wellbeing, quality of life and identity [9, 32], in some individuals a more negative impact than the diagnosis of cancer itself [39]. Communication about fertility with HCPs can alleviate patient distress and enable coping, as well as being the first step in the process of fertility preservation, where this is appropriate. Information about fertility is thus undoubtedly “a crucial aspect of high quality healthcare” in the context of cancer [14], and cannot be overlooked.

## Abbreviations

AYA: Adolescent and Young Adult
HCPs: Health Care Professionals
LGBTI: Lesbian, Gay, Bisexual, Transgender, Intersex
